# Physicochemical, sensory, and antioxidant characteristics of stirred‐type yogurt enriched with *Lentinula edodes* stipe powder

**DOI:** 10.1002/fsn3.3563

**Published:** 2023-07-19

**Authors:** Hanyu Zhu, Zheng Chen, Geqing Li, Xiaoqian Yao, Yujing Hu, Wenxia Zhao

**Affiliations:** ^1^ College of Life Science Hengyang Normal University Hengyang China; ^2^ Hunan Key Laboratory for Conservation and Utilization of Biological Resources in the Nanyue Mountainous Region Hengyang Normal University Hengyang China; ^3^ College of Nanyue Hengyang Normal University Hengyang China; ^4^ Xinjiang Seed Industry Development Center of China China

**Keywords:** antioxidant activity, LES, physicochemical property, sensory evaluation, stirred yogurt

## Abstract

The *Lentinula edodes* stipe (LES), a by‐product of *L. edodes* fruiting body processing, is rich in dietary fiber, protein, and polysaccharides, which can be served as the functional ingredient in dairy products. In this study, stirred yogurts fortified with 1%, 2%, and 3% LES were prepared, and the effects of LES on the changes in color, pH, titratable acidity (TA), viable lactic acid bacteria (LAB) cells, syneresis, viscosity, texture, and antioxidant activity of the flavored yogurt were monitored at the beginning and the end of storage. The LES decreased the lightness, increased the red–green color values and yellow–blue color values, decreased the pH values, and increased the contents of TA, the viable LAB cells, and the antioxidant activity of yogurt samples in a dose‐dependent manner. The addition of LES showed double‐edged effects on the texture of yogurt, which significantly reduced firmness and viscosity but decreased the syneresis. Compared with plain yogurt, the 2% LES‐fortified yogurt exhibited similar index values of texture parameters and higher scores of the appearance, fermented odor, taste quality, and overall acceptance, suggesting that this might be the optimal dose for industrial production. After cold storage for 28 days, pH values of all yogurt samples further decreased with increasing of TA. Interestingly, syneresis of LES‐fortified yogurt decreased and the viable LAB cells and antioxidant activity of 3% LES‐fortified yogurt slightly decreased. Therefore, LES is beneficial to improve physicochemical, sensory, and antioxidant properties of yogurt, which has the potential to be used in functional dairy products.

## Graphical abstract

According to the methodical examination of the physicochemical, sensory, and antioxidant properties of flavored yogurts which fortified with different amounts of *Lentinula edodes* stipe (LES), we found that the LES was beneficial to improve some physicochemical (pH, viable lactic acid bacteria density, and syneresis) and sensory properties (fermented odor and taste quality), and the antioxidant activity (ABTS and DPPH radical scavenging activity) of the plain yogurt, which has the potential to be used in functional yogurt. The results above were also helpful for the rational utilization of LES toward value‐added products.

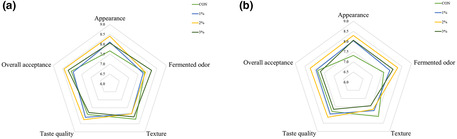

## 1 INTRODUCTION

*Lentinus edodes* (LE) is the second‐most popular and cultivated edible mushroom worldwide due to its tasty flavor, high nutritional value, and considerable health‐promoting properties (Lu et al., 2023). It is not only considered as a reliable source of protein, vitamins, fatty acids, and several microelements but also contains a variety of bioactive substances, such as dietary fiber, polysaccharides, polyphenols, and ergosterol (Lu, Wang, et al., 2022; Tian et al., 2022). Among them, lentinan, the LE‐derived β‐glucans, is a most well‐studied and widely approved polysaccharide medicine which has been demonstrated to have immunomodulating, antioxidant, antitumor, and anticancer properties (Wang et al., 2021; Zhang et al., 2022).

In China, LE has a huge annual production of more than 11.88 million tons (Zong et al., 2022). A complete LE consists of two parts, the cap and the stipe, and *Lentinus edodes* stipe (LES) usually accounts for about 25%–33% of the total weight (Chou et al., 2013). It has been shown that the contents of dietary fiber, carbohydrate, and calcium in stipes were higher than those in caps of LE which makes LES become an attractive and promising source of dietary ingredients (Li et al., 2018; Sari et al., 2017). However, LES was usually considered to be a waste product during harvest, probably because of their undesirable sensory characteristics to consumers (Harada‐Padermo et al., 2020). According to statistics, nearly three million tons of LES were discarded and wasted every year merely in China (Lu et al., 2023). Combined with the rapid development of the edible fungi industry in the world, the resource utilization of their by‐products has become an urgent problem for the industry's sustainable development (Tian et al., 2022), and LES, thus, deserves more attention for rational utilization toward value‐added products.

Yogurt, the most popular fermented dairy product consumed worldwide, contains nutritive functions and beneficial actions in the reduction of gastrointestinal discomfort, eliminating symptoms of lactose intolerance, strengthening the immune system, protection against colon cancer and *Helicobacter pylori* infection, and elevating metabolic rates, thereby presenting it as an essential ingredient of healthy diets (Helal et al., 2022). However, evidence suggests that plain yogurts do not possess large quantities of bioactive compounds (Dimitrova‐Shumkovska et al., 2022). On the other hand, low viscosity and high syneresis are the primary defects of yogurt which play an essential role in consumer acceptability (Kim et al., 2019). Many studies have demonstrated that the fortification of yogurt using natural resources could improve the texture and functionality of yogurt with minimal adverse effects (Du et al., 2021; Helal et al., 2022; Hong et al., 2020). Given the current popularity of yogurt consumption as seen from the global market profits, as well as experimental evidences of nutritional values and bioactivity of LES, the LES‐fortified yogurt will increase the interest in the manufacture of dairy products.

In this study, we aimed to methodically examine the physicochemical, sensory, and bioactivity properties of flavored yogurts which fortified with different amounts of LES. Yogurts were prepared by incorporating LES powder into stirred yogurt and changes in the color, pH, total acid, rheological properties, texture, sensory, and antioxidant capacities related to the LES proportion and storage time were monitored in order to illuminate the effect of LES on the fortified yogurt.

## 2 MATERIALS AND METHODS

### 2.1 Materials

The *Lentinula edodes* stipe (LES) was dried by hot air at 60°C and then at 37°C until a constant weight was achieved. Dried LES was ground using a plant pulverizer and passed through a 120‐mesh sieve to obtain LES powder, which was stored under −18°C for later use. Contents of crude polysaccharides, protein, fat, and ash of LES were 79.69 ± 0.43, 14.58 ± 0.39, 1.55 ± 0.13, and 4.17 ± 0.02 g/100 g (dry weight, DW) according to the Association of Official Analytical Chemists methods (Hasan, 2015), and the total phenolic content of LES aqueous extract was 7.40 ± 0.17 mg gallic acid equivalent (GAE)/g DW. The starter culture (containing *Streptococcus thermophilus* and *Lactobacillus bulgaricus*, Angel Yeast, Hubei, China) and market cow milk containing 3.2% of milk protein (Mengniu, Inner Mongolia, China) were purchased from a local supermarket. The reagents were of analytical grade or authentic standard chemicals.

### 2.2 Yogurt preparation

The market cow milk and sucrose (8%, g/mL) were mixed and subsequently homogenized for 5 min. Thereafter, the homogenate was sterilized for 30 min at 85°C, then cooled to 42°C. The cooled milk was inoculated with 1 g/L of starter culture, fermented in an incubator at 42°C for 6 h of curdling time, and then cooled to 10°C. The LES‐enhanced flavored yogurt was prepared by adding 1%, 2%, and 3% (wt/wt) LES powder to the cooled plain yogurt, respectively. After stirring gently for 3 min, fortified flavored yogurt was distributed and stored in a dark environment at 4°C for 28 days. Characteristics and qualities of the samples were evaluated in triplicate at the storage time of 1 and 28 days.

### 2.3 Physicochemical and microbiological determinations

The pH, titratable acidity (TA), color, and LAB density of the yogurts were measured at the refrigerated storage time of 1 and 28 days. The pH was measured using a pH meter (Ohaus, New Jersey, USA). The TA was determined following the method described by Cho et al. (2020). A well‐mixed homogeneous 10‐g yogurt sample was diluted with 20 mL distilled water and two to three drops of phenolphthalein were added as indicator. Then, it was titrated against 0.1 mol/L NaOH solution until a pink color appears as the end point which was retained for 30 s. The percentage of acidity was calculated as follows: TA (%) = (0.009 × volume of NaOH used × 0.1/weight of yogurt sample) × 100.

The color of fortified flavored yogurt was measured using a colorimeter (Konica, Minolta, Chroma, Meter CR‐400). The parameters of lightness value (L*), red–green value (a*), and yellow–blue value (b*) were reported. The L* value ranges from 0 (black) to 100 (white). Coordinate a* represents red (positive) to green (negative) and b* represents yellow (positive) to blue (negative). Before testing, the device was calibrated on a white reference standard.

The LAB density was determined using the streak plate method with MRS agar medium. Samples were gradient diluted 10 times with 0.9% NaCl solution. After spreading and smearing the diluted solution (100 μL) onto MRS agar plates, they were cultured at 37°C for 48 h. The total number of viable cells was expressed as a log‐transformed value.

### 2.4 Rheological measurements

The viscosity, susceptibility to syneresis, and texture analysis of yogurts were estimated at the refrigerated storage time of 1 and 28 days. The viscosity was measured on a cup at 4°C with a viscometer (DV‐II, Brookfield, Middleboro, MA, USA). The spindle used (spindle no. 3 at 30 rpm) in 150 g of yogurt was allowed to rotate for 1 min.

Syneresis of yogurt was determined by centrifuging 20 g of samples at 5000 *× g* for 5 min at 4°C and weighing the supernatant. Percent syneresis was calculated as: Syneresis (%) = weight of supernatant (g)/weight of sample (g) × 100%.

The texture analysis was determined by a TAXT Texture Analyzer (Stable Micro System) with a backward extrusion test. The cylindrical probe diameter of 36 mm was used for the purpose. Pretest speed, test speed, posttest speed, trigger force, and distance were 1.0 mm/s, 1.0 mm/s, 2.0 mm/s, 10.0 g, and 10.0 mm, respectively, as described by Du et al. (2021). The diameter of beaker for holding the sample was large enough to minimize the probe side wall effects. The firmness (N), consistency (N × s; total positive area), cohesiveness (N; maximum adhesive force), and viscosity index (N × s; total negative area) were calculated using texture exponent software (Exponent, version 6.11.16.0, Stable Micro Systems).

### 2.5 Sensory evaluation

Twenty semitrained panelists who were trained following the procedure of Meilgard et al. (2015) were asked to evaluate the sensory attributes of the yogurts. These panelists (10 women and 10 men, aged between 18 and 40) include staff members, graduate and undergraduate students of the Department of Food Science and Technology, Hengyang Normal University. Before sensory evaluation, samples were taken out from the refrigerator and served to panelists immediately (Kaur & Riar, 2020). The ratings were presented on a 9‐point hedonic ranking scale with the following score expressions: 1 = highly dislike, 2 = dislike very much, 3 = rather dislike, 4 = dislike a little, 5 = neither like nor dislike, 6 = quite like, 7 = rather like, 8 = like a lot, 9 = like very much (Sheikh et al., 2022). Yogurt sensory parameters were evaluated by appearance, fermented odor, texture, taste quality, and overall acceptance. The values obtained for each sensory perception were given on averages of a number of panelists' values in duplicate for each sample.

### 2.6 Antioxidant activity assay

All yogurt samples were applied to estimate the antioxidant activity. The 1,1‐diphenyl‐2‐picrylhydrazyl (DPPH) and 2,2′‐Azino‐bis (3‐ethylbenzothiazoline‐6‐sulfonic acid) diammonium salt (ABTS) radical scavenging activity was measured according to the previous method (Xu et al., 2019) with slight modification. Briefly, the water‐soluble extracts were prepared by first mixing 10 g of yogurt samples in distilled water (100 mL) followed by a thorough shake for a period of 2 min and were collected by centrifugation at 5000 × *g* for 10 min. Furthermore, 50‐μL sample solution was mixed with 150 μL DPPH‐ethanol or ABTS solution. Then, the mixture was incubated in the dark for 30 min at room temperature, and the absorbance at 517 nm or 734 nm was measured using a spectrophotometric microplate reader. Different concentrations of L‐ascorbic acid varying from 0 to 30 μg/mL were then used to prepare a standard curve and the antioxidant properties of samples were expressed as microgram of ascorbic acid per gram of dry weight extract (μg ascorbic acid/g dw) (Khatua & Acharya, 2022).

The total phenolic content (TPC) of yogurt samples was determined by the Folin–Ciocalteu assay according to the method of Sheikh et al. (2022). A gallic acid solution with concentrations of 0–100 μg/mL was used to construct the calibration curve and was treated in the same way as the sample solutions. The values of TPC were expressed as the GAE per gram of yogurt samples.

### 2.7 Statistical analysis

All experiments were carried out in triplicate. Results were shown as means ± standard deviation. Statistical significance was determined by one‐way analysis of variance (ANOVA) and performed using SPSS 23.0 software (SPSS Inc.). Duncan's post hoc test and Student's *t*‐test were applied to test significant differences with the significance level. Values of *p* < .05 were considered statistically significant.

## 3 RESULTS AND DISCUSSION

### 3.1 Color parameters

**FIGURE 1:**
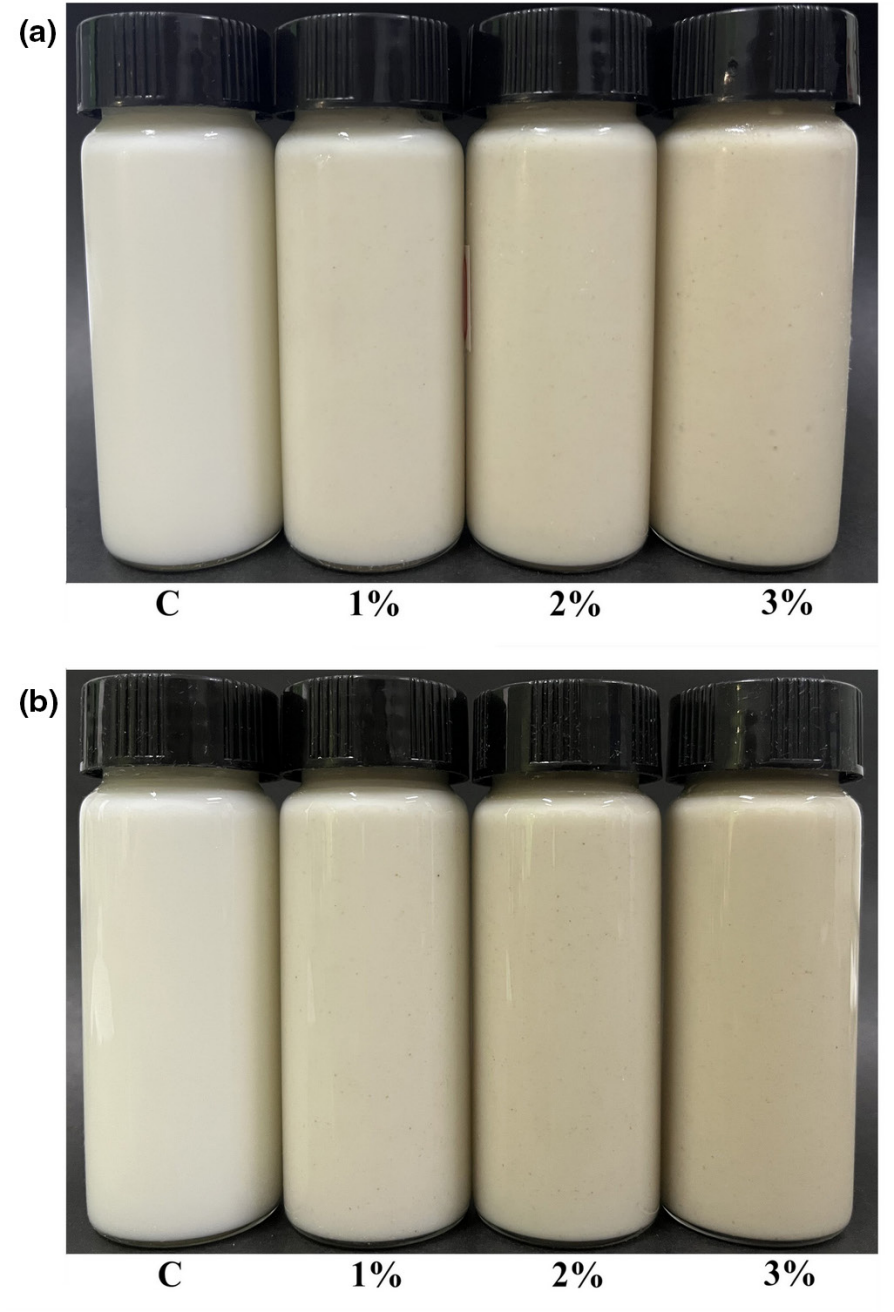
Appearance of yogurt samples with different amounts of *Lentinula edodes* stipe (LES) powder added which were stored for 1 day (a) and 28 days (b). C: the plain yogurt (as the control); 1%: 1% LES‐fortified yogurt; 2%: 2% LES‐fortified yogurt; 3%: 3% LES‐fortified yogurt.

Yogurt is a popular dairy product with numerous health benefits and functional properties (Helal et al., 2022). In this study, the stirred yogurts fortified with 1%, 2%, and 3% LES were prepared and compared with the plain yogurt which was used as the control. After fermentation, the yogurts were further stored at 4°C for 28 days. For yogurt quality, color is an important parameter which has a substantial influence on acceptance by consumers (Kim et al., 2019). We found that the product showed light yellow after adding LES powder (Figure 1). The color parameters of the stirred flavored yogurt samples supplemented with different amounts of LES were significantly different (*p* < .05, Table 1). The L* values (lightness) of the three groups of LES‐fortified yogurt significantly decreased, whereas their a* and b* values significantly increased (*p* < .05) when compared with the control. After being stored for 28 days, the color of LES‐fortified yogurt samples kept relatively constant, although their L*, a*, and b* showed a decreasing tendency (Table 1). Our results were similar to those of flavored yogurts supplemented with other natural materials such as mulberry pomace (Du et al., 2021), paprika juice (Hong et al., 2020), and lotus leaf (Kim et al., 2019).

**TABLE 1:** Color analysis of the stirred yogurt incorporated *Lentinula edodes* stipe (LES) at the refrigerated storage time of 1 and 28 days.

Treatment	Parameter^1^	Storage time (d)		*p*‐Value^2^
		1	28	
C	L*	91.24 ± 2.00^a^	94.44 ± 0.44^a^	.103
	a*	−1.71 ± 0.03^d^	−1.86 ± 0.09^d^	.041
	b*	7.96 ± 1.06^c^	7.75 ± 0.26^d^	.771
1%	L*	84.48 ± 2.16^b^	81.29 ± 1.21^b^	.089
	a*	0.99 ± 0.04^c^	0.84 ± 0.06^c^	.019
	b*	13.48 ± 0.53^b^	10.74 ± 0.29^c^	.001
2%	L*	77.34 ± 1.49^c^	76.49 ± 0.20^c^	.424
	a*	2.32 ± 0.17^b^	2.20 ± 0.02^b^	.290
	b*	14.89 ± 0.16^a^	12.94 ± 0.04^b^	.000
3%	L*	74.36 ± 4.90^c^	71.91 ± 0.95^d^	.479
	a*	3.02 ± 0.23^a^	3.16 ± 0.10^a^	.394
	b*	15.59 ± 0.21^a^	14.44 ± 0.05^a^	.001

*Note*: All values are means ± SD (*n* = 3). Different letter superscripts indicate statistically significant differences (*p* < .05) between the yogurts fortified with different amounts of LES (one‐way analysis of variance followed by Duncan's post hoc test). C: the plain yogurt (as the control); 1%: 1% LES‐fortified yogurt; 2%: 2% LES‐fortified yogurt; 3%: 3% LES‐fortified yogurt.

^1^ L* = lightness; a* = red–green color; b* = yellow–blue color.

^2^ *p*‐Values indicate statistical differences between the yogurts throughout different points of storage (Student's *t*‐test).

### 3.2 pH, TA, and LAB density

**FIGURE 2:**
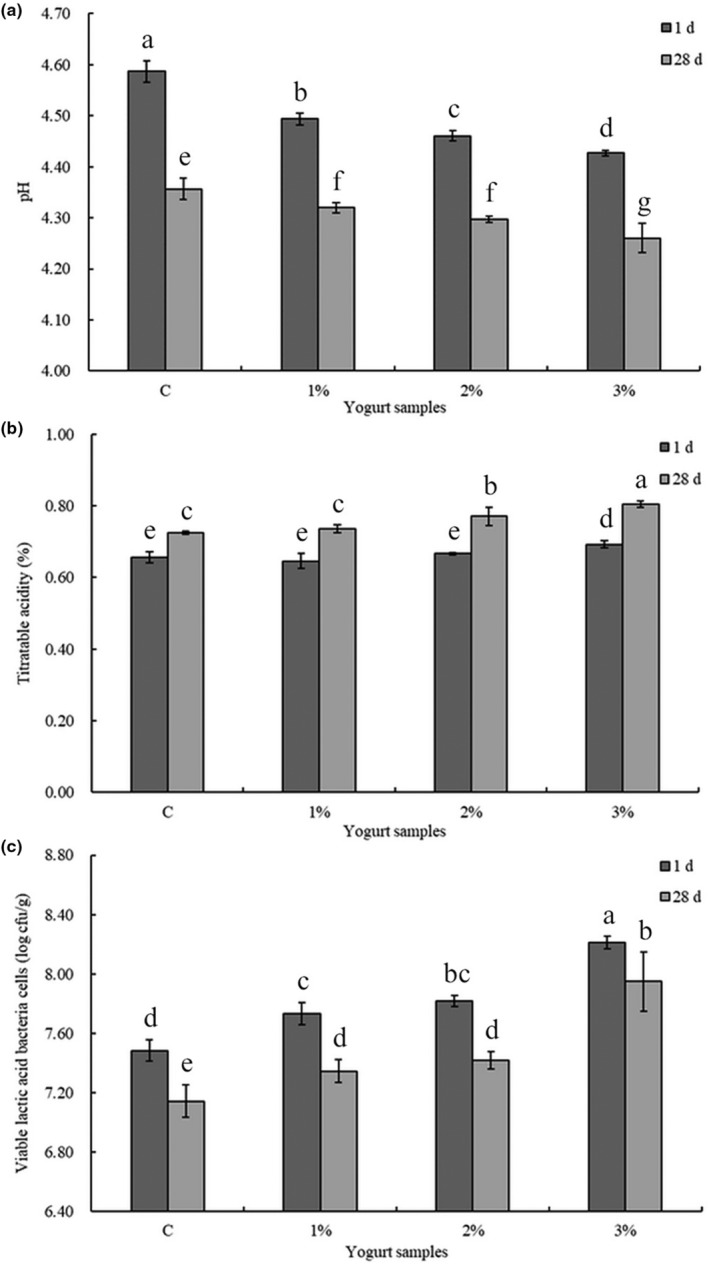
The pH (a), titratable acidity (b), and viable lactic acid bacteria cells (c) of stirred yogurt containing *Lentinula edodes* stipe (LES) powder when stored at 4°C. C: the plain yogurt (as the control); 1%: 1% LES‐fortified yogurt; 2%: 2% LES‐fortified yogurt; 3%: 3% LES‐fortified yogurt. Different lowercase letters above the columns indicate statistically significant differences (*p* < .05) between the yogurts fortified with different amounts of LES (one‐way analysis of variance followed by Duncan's post hoc test).

At the beginning of storage time (1 day), pH values of yogurt samples decreased with the increasing addition of LES, and their TA increased accordingly (*p* < .05, Figure 2), which might be accounted for the organic acids in LES powder, including acetic acid, citric acid, fumaric acid, and malic acid (Chen et al., 2015; Wen et al., 2022). At the end of storage time (28 days), the decreased pH values and increased TA of all yogurt samples were observed (Figure 2) which is due to the slow fermentation of LAB during the storage (Du et al., 2021), and the highest TA value occurred in the 3% LES‐fortified yogurt. Nutrients such as polysaccharides and polyphenol compounds in LES added to the yogurt could be served as prebiotics for LAB, and probably caused the additional production of lactic acid and other organic acids (Sharma & Padwad, 2020).

According to FAO/WHO, the total viable number of LAB in fermented beverages should exceed 7 log cfu/g (Dimitrellou et al., 2019). However, increasing acidity and oxidative pressure will decrease the viable number of LAB during the storage periods of dairy products (Chou et al., 2013). Consequently, ensuring the viability of probiotics during fermentation and storage is a critical issue in the development of yogurt products, and high numbers of viable probiotic microorganisms in yogurts could easily fulfill the demand to accomplish a probiotic action in the host (Jovanović et al., 2020). The LAB densities in the current study were above the minimum requirement in all groups of yogurts. At the storage time of 1 day, viable LAB counts in the control and 1%, 2%, and 3% LES‐fortified yogurt samples were 7.48 ± 0.07, 7.73 ± 0.07, 7.82 ± 0.04, and 8.21 ± 0.04 log cfu/g (*p* < .05, Figure 2c), respectively. It seemed that high LES dosage could promote the survival rate of LAB, which was further corroborated by the viable LAB counts in 3% LES‐fortified yogurt samples at the end of storage. The number of LAB cells in 3% LES‐fortified yogurt sample was just slightly decreased without significant differences (*p* > .05) after refrigerated storage for 28 days and was significantly higher than those in the control (*p* < .05). It has been reported that the polysaccharides had synergistic effects with the peptides and amino acids from a yogurt culture to maintain LAB, and they also had significant protective effects on these probiotics in simulated gastric and bile juice conditions to achieve beneficial effects in the host (Chou et al., 2013). These results showed that LES could be used as an important, new, alternative source of prebiotics in maintaining probiotics.

### 3.3 The structure and rheological properties

**FIGURE 3:**
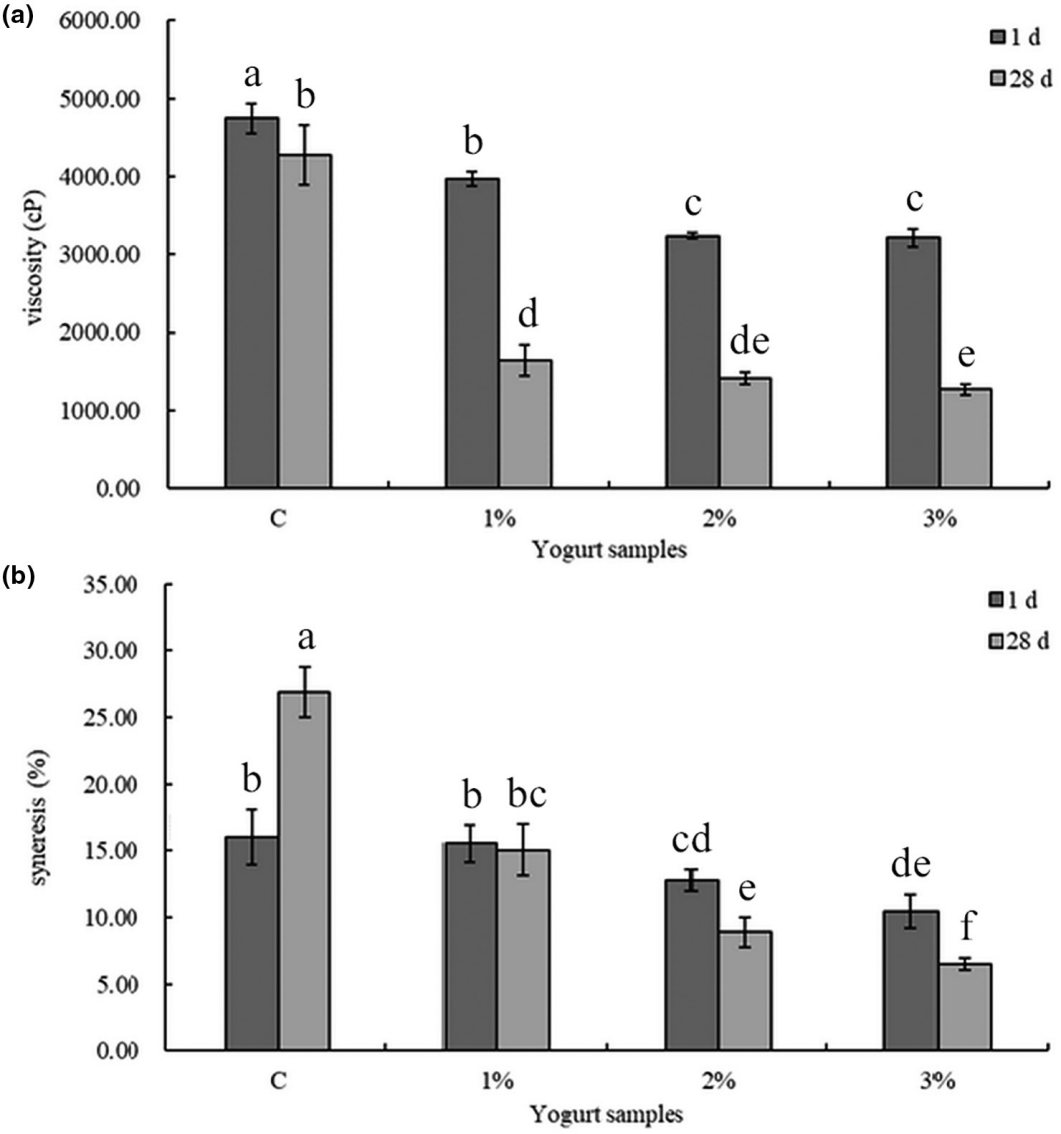
The viscosity (a) and syneresis (b) values of stirred yogurt fortified with *Lentinula edodes* stipe (LES) powder when stored at 4°C. C: the plain yogurt (as the control); 1%: 1% LES‐fortified yogurt; 2%: 2% LES‐fortified yogurt; 3%: 3% LES‐fortified yogurt. Different lowercase letters above the columns indicate statistically significant differences (*p* < .05) between the yogurts fortified with different amounts of LES (one‐way analysis of variance followed by Duncan's post hoc test).

The structure and the rheological properties of yogurt are important to the yield, sensory evaluation, stability, texture, and shelf life (Jovanović et al., 2020; Kaur & Riar, 2020). Values of viscosity and syneresis of all yogurt samples are shown in Figure 3. The addition of LES significantly reduced the viscosity value in a concentration‐dependent manner (*p* < .05, Figure 3a). These results were consistent with previous reports that the viscosity of yogurt decreased with increasing addition of mulberry pekmez (Celik & Bakirci, 2003), black garlic extract (Shin et al., 2010), cherry pulp (Sengul et al., 2012), or fermented red or green pepper juice (Kang et al., 2018). It is probably because the addition of LES promoted the growth of LAB and thereby decreased the gel strength by promoting degradation of milk solid components or pH‐induced changes in casein micelles (Kang et al., 2018), resulting in the lower viscosity of yogurt. Thus, researches on improving the structure of LES‐fortified yogurt by using stabilizing agents such as starches, gelatine, or pectin will be taken into consideration in our future work.

Syneresis, causing watery whey‐like liquid on the surface of the yogurt is regarded as a technological defect in yogurt (Dönmez et al., 2017). Syneresis of the 1%, 2%, and 3% LES‐fortified yogurts showed decreasing tendency and had significantly lower syneresis values (*p* < .05) on both 1 and 28 days compared with the control (Figure 3b). The decreasing syneresis of LES‐fortified yogurts was possibly attributed to the strong affinity between polyphenols of LES and dairy proteins, which could obstruct whey separation (Dimitrova‐Shumkovska et al., 2022). Polyphenols of LES could be revealed by the TPC of LES aqueous extract which was 7.40 ± 0.17 mg gallic acid equivalent (GAE)/g DW, and it was higher than those in *Ganoderma lucidum* (Kebaili et al., 2021), *Cantharellus cibarius* (Ghosh et al., 2023), *Phellinus Igniarius*, and *P. torulosus* (Dimitrova‐Shumkovska et al., 2022). In addition, polysaccharides or dietary fiber, as hydrocolloids to trap free water and facilitates the link with water molecules in yogurts, also have the ability to reduce susceptibility to syneresis (Du et al., 2021). Interestingly, the syneresis was decreased in the LES‐fortified samples during storage, whereas increased significantly in the control (*p* < .05). The decreased syneresis was consistent with the decline of TPC in LES‐fortified yogurts which might be because of the increased interactions between milk proteins and polyphenols during storage (Hamed et al., 2021) and thereby lowered syneresis. A similar decreasing pattern was also registered in the *P. Igniarius* and *P. torulosus* added yogurts during storage time of 14 days (Dimitrova‐Shumkovska et al., 2022). These specific functional properties of LES may be useful for improving the syneresis of yogurt and deserves further study to explain it.

From the texture profile analysis, the LES‐fortified yogurt exhibited lower firmness and viscosity index, similar cohesiveness, and higher consistency compared with the control (*p* < .05, Table 2). Generally, firmness describes moderate resistance of product to deformation and cohesiveness is the tendency of a product to cohere or stick together, while consistency relates to the “firmness”, “thickness”, or “viscosity” of a liquid or fluid semisolid (Hovjecki et al., 2020). The addition of LES decreased the firmness of yogurt in a dose‐depend manner, which probably accounted for the lower syneresis. It led to the higher water content of the yogurt sample, thus resulting in increased softness and then further decrease firmness of yogurt. The addition of LES had little effect on the cohesiveness of yogurt, especially at 28 days (*p* > .05). The result of viscosity index was consistent with the viscosity data, showing intensified trend with decreasing LES level. Except for consistency, values of all the texture parameters of LES‐fortified yogurt decreased after storage for 28 days. The 2% LES‐fortified yogurt exhibited similar index values of firmness, cohesiveness, consistency, and viscosity compared with the control, which suggested that this might be the optimal dose for industrial production.

**TABLE 2:** Texture profile analysis of *Lentinula edodes* stipe (LES)‐fortified yogurt at the refrigerated storage time of 1 and 28 days.

Parameter	Treatment	Storage time (d)		*p*‐Value*
		1 day	28 days	
Firmness (g)	C	10.97 ± 0.43	10.06 ± 1.44^a^	.356
	1%	10.81 ± 0.50	6.79 ± 0.10^b^	.000
	2%	10.67 ± 0.21	6.71 ± 0.35^b^	.000
	3%	10.92 ± 0.38	6.22 ± 1.07^b^	.002
Consistency (g × s)	C	0.35 ± 0.07^ab^	0.55 ± 0.08^a^	.030
	1%	0.27 ± 0.01^b^	0.31 ± 0.01^b^	.013
	2%	0.31 ± 0.01^ab^	0.31 ± 0.01^b^	.518
	3%	0.39 ± 0.07^a^	0.49 ± 0.07^a^	.125
Cohesiveness (g)	C	19.03 ± 1.48^a^	14.77 ± 2.99	.091
	1%	24.75 ± 0.70^b^	14.94 ± 0.89	.000
	2%	24.00 ± 1.37^b^	16.49 ± 0.59	.001
	3%	17.32 ± 1.21^a^	16.09 ± 4.31	.658
Viscosity index (g × s)	C	2.66 ± 1.04	1.78 ± 0.35^b^	.237
	1%	2.55 ± 0.15	1.71 ± 0.09^b^	.001
	2%	2.58 ± 0.33	1.54 ± 0.19^ab^	.010
	3%	2.51 ± 0.16	1.14 ± 0.20^a^	.001

*Note*: All values are means ± SD (*n* = 3). Different letter superscripts indicate statistically significant differences (*p* < .05) between the yogurts fortified with different amounts of LES (one‐way analysis of variance followed by Duncan's post hoc test). C: the plain yogurt (as the control); 1%: 1% LES‐fortified yogurt; 2%: 2% LES‐fortified yogurt; 3%: 3% LES‐fortified yogurt.

* *p*‐Value indicates statistical differences between the yogurts throughout different points of storage (Student's *t*‐test).

### 3.4 Antioxidant assays

Free radicals are reactive oxygen species in cells, constantly produced in the human body, which can cause DNA mutation, protein damage, lipid peroxidation, and several chronic and degenerative diseases including Alzheimer's, Parkinson's, diabetes, and cancer (Xu et al., 2019). It is a common scientific perception that the bioactive compounds such as polyphenols, polysaccharides, and flavonoids contained in mushrooms are the main contributors to their in vitro antioxidant efficiency (Sułkowska‐Ziaja et al., 2018). Usually, the antioxidant capacity is determined by complicated factors with various action mechanisms, and it is recommended that at least two methods of antioxidant activity should be evaluated in foods (Rosa et al., 2020). Therefore, the antioxidant activity of LES‐fortified yogurts was examined through DPPH and ABTS radical scavenging assays. The unstable DPPH radical (purple) can accept an electron or a hydrogen radical donated by antioxidants to become a stable nonradical form DPPH‐H (yellow), and the formation of ABTS radical (blue–green) can be prevented under the presence of antioxidants which results in decrease in absorption (Khatua & Acharya, 2022).

Herein, the antioxidant capacity of yogurts was expressed as ascorbic acid (widely used as a standard to evaluate antioxidant activity) equivalents (AAE). As summarized in Table 3, the AAE values with ABTS and DPPH assays of all yogurt samples ranged from 0.45 to 2.64 mg ascorbic acid/g dw, and from 0.50 to 2.77 mg ascorbic acid/g dw. It can be observed that the addition of LES increased the antioxidant capacity of yogurts in a concentration‐dependent manner, and the 3% LES‐fortified yogurt showed the highest ABTS and DPPH radical scavenging activity at the storage time of 1 and 28 days (*p* < .05). The control always presented the lowest antioxidant capacity. Corroborating the results presented here, previous studies demonstrated that the polysaccharides from LES had been proved to have the DPPH and ABTS radical scavenging rate reached 43.58% and more than 90% when the concentration was 2 mg/mL (Li et al., 2019).

**TABLE 3:** Antioxidant assays and total phenolic content of *Lentinula edodes* stipe (LES)‐fortified yogurt at the refrigerated storage time of 1 and 28 days.

Antiradical assays	Treatment	Storage time (d)		*p*‐Value*
		1 day	28 days	
AAE_ABTS_ (μg ascorbic acid/g FW)^a^	C	1006.96 ± 92.65^d^	445.74 ± 21.00^d^	.001
	1%	1639.98 ± 137.26^c^	935.02 ± 17.49^c^	.001
	2%	2052.20 ± 37.26^b^	1605.65 ± 76.74^b^	.001
	3%	2635.82 ± 45.50^a^	2613.65 ± 24.91^a^	.500
AAE_DPPH_ (μg ascorbic acid/g FW)^b^	C	2393.36 ± 60.61^b^	501.08 ± 61.25^d^	.000
	1%	2459.46 ± 132.89^b^	1065.09 ± 204.75^c^	.001
	2%	2458.75 ± 112.43^b^	1818.81 ± 83.58^b^	.001
	3%	2773.63 ± 83.24^a^	2244.76 ± 136.17^a^	.005
TPC (μg gallic acid/g FW)^c^	C	102.29 ± 1.50^d^	38.93 ± 0.10^d^	.000
	1%	199.50 ± 3.20^c^	150.45 ± 2.00^c^	.000
	2%	281.06 ± 9.42^b^	195.07 ± 1.56^b^	.003
	3%	331.20 ± 2.09^a^	228.31 ± 4.42^a^	.000

*Note*: All values are means ± SD (*n* = 3). Different letter superscripts indicate statistically significant differences (*p* < .05) between the yogurts fortified with different amounts of LES (One‐way Analysis of Variance followed by Duncan post hoc test). C: the plain yogurt (as the control); 1%: 1% LES‐fortified yogurt; 2%: 2% LES‐fortified yogurt; 3%: 3% LES‐fortified yogurt.

* *p* Values indicate statistical differences between the yogurts throughout different points of storage (Student's *t*‐test).

^a^ Ascorbic acid equivalent antioxidant capacity (AAE), 2,2′‐Azino‐bis (3‐ethylbenzothiazoline‐6‐sulfonic acid) diammonium salt (ABTS), fresh weight (FW).

^b^ 1,1‐diphenyl‐2‐picrylhydrazyl (DPPH).

^c^ Total phenolic content (TPC).

^d^ The ‘d’ means significantly difference from ‘a’, ‘b’ and ‘c’ in the same column of same parameters.

In addition, polyphenols from the aqueous and ethanolic extracts of LE had relatively high amounts and were determined to be the major antioxidant component (Choi et al., 2016; Finimundy et al., 2013). Because of the hydroxyl substituents and aromatic structures, polyphenols have strong antioxidant capacities (Kim et al., 2019), and the yogurt matrix appears to be an effective vehicle for the delivery of phenolic compounds (Helal et al., 2022). From the results of our study (Table 3), LES powder significantly influenced the TPC in yogurt in a concentration‐dependent manner (*p* < .05), showing a positive effect on the antioxidant potency of dairy products which was consistent with previous reports (Dimitrova‐Shumkovska et al., 2022). What is more, the TPC of all samples was significantly decreased during the storage, which might be due to the increased interactions between milk proteins and polyphenols (Kim et al., 2019) and the decomposition of polymeric phenolics in the presence of lactic acid bacteria during refrigerated storage (Hamed et al., 2021). Thereafter, the antioxidant activity of yogurt samples was decreased accordingly at the end of storage.

### 3.5 Sensory analysis

**FIGURE 4:**
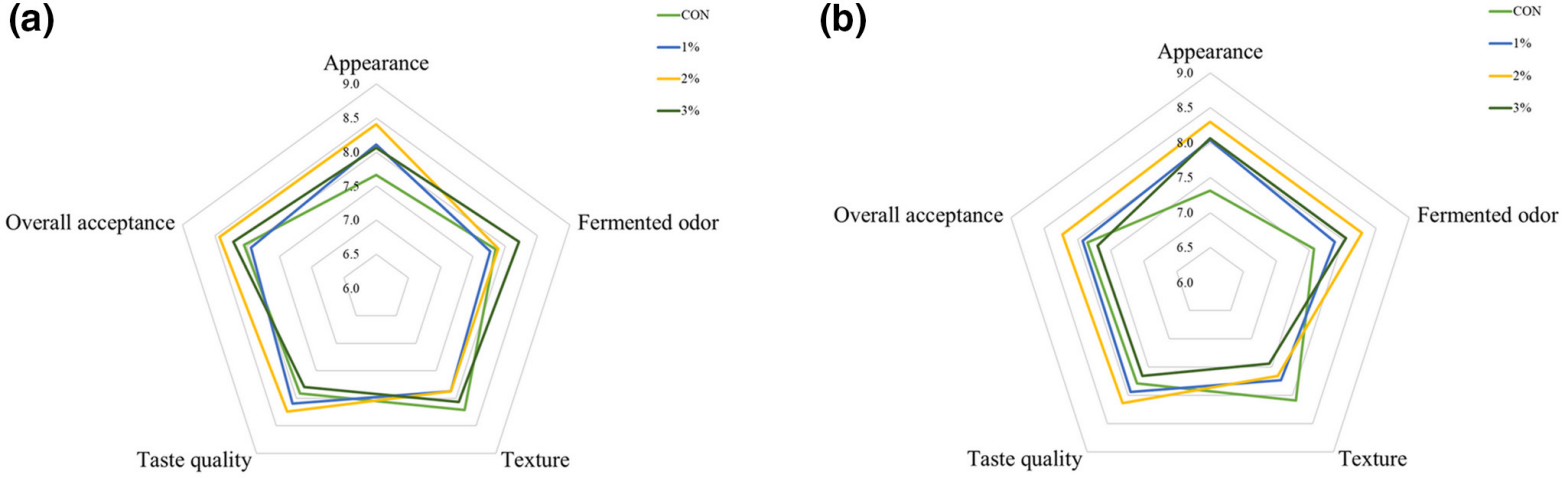
Sensory analysis of stirred yogurt fortified with *Lentinula edodes* stipe (LES) powder when stored at 4°C for 1 day (a) and 28 days (b). C: the plain yogurt (as the control); 1%: 1% LES‐fortified yogurt; 2%: 2% LES‐fortified yogurt; 3%: 3% LES‐fortified yogurt.

The sensory evaluation was conducted for the yogurt samples at the storage time of 1 and 28 days (Figure 4). The maximum values of overall acceptance were obtained for 2% LES‐fortified yogurt with statistically significant (*p* < .05) differences, which were 8.43 ± 0.46 at 1 day and 8.23 ± 0.28 at 28 days. In the case of texture of yogurt, viscosity and firmness were the main parameters, and the samples containing LES had significantly lower scores that the control, which was in accordance with the lower viscosity and firmness in LES‐fortified yogurt. It is worth mentioning that addition of LES significantly improved the fermented odor and taste quality of yogurt (*p* < .05). In dried LE, volatile sulfur–containing compounds, eight‐carbon compounds, and aldehydes had great contributions to its distinctive flavor (Lu, Hou, et al., 2022), and LES were rich in tasty components, including soluble sugars, free amino acids, 5′‐nucleotides, and organic acids, which contributed to a variety of taste, such as sweet, umami, sour, and so on (Chen et al., 2015). These compounds might have a positive effect on the fermented odor and taste quality of LES‐fortified yogurt.

## 4 CONCLUSIONS

The results of current study showed that stirred yogurt fortified with LES had decreased lightness, pH values, and viscosity, increased red–green color values and yellow–blue color values, contents of TA and viable LAB cells, ABTS and DPPH radical scavenging activities in a dose‐dependent manner both at the storage time of 1 and 28 days. The addition of LES reduced firmness and viscosity index, which might be accounted for lower scores of the texture in sensory analysis and stabilizing agents such as starches, gelatine, or pectin could be applied for improving the structure of LES‐fortified yogurt. According to texture and sensory analyses, the 2% LES‐fortified yogurt exhibited similar index values of firmness, cohesiveness, consistency, viscosity and higher scores of the appearance, fermented odor, taste quality, and overall acceptance compared with the control, suggesting that this might be the optimal dose for industrial production. Therefore, yogurt containing LES with improved physicochemical, sensory, and antioxidant properties could be a healthy dietary product to meet the demand for new functional foods and the trend of sustainable development of edible mushroom by‐products.

## AUTHOR CONTRIBUTIONS

**Hanyu Zhu:** Conceptualization (equal); funding acquisition (lead); investigation (equal); supervision (equal); writing – original draft (equal). **Zheng Chen:** Conceptualization (equal); data curation (equal); supervision (equal). **Geqing Li:** Data curation (equal); methodology (equal); writing – original draft (equal). **Xiaoqian Yao:** Data curation (equal); investigation (equal); methodology (equal). **Yujing Hu:** Methodology (equal). **Wenxia Zhao:** Writing – review and editing (lead).

## CONFLICT OF INTEREST STATEMENT

The authors declare that they have no conflict of interest.

## Data Availability

All relevant data are within the paper and are available upon request from the authors.
